# Mapping the relationship between alcohol use disorder and gut microbiota: a 20-year bibliometric study

**DOI:** 10.3389/fmicb.2024.1457969

**Published:** 2024-11-18

**Authors:** Ao Xiang, Yonglong Chang, Li Shi, Xuhui Zhou

**Affiliations:** ^1^Department of Psychiatry, The School of Clinical Medicine, Hunan University of Chinese Medicine, Changsha, China; ^2^Department of Psychiatry, The Second People’s Hospital of Hunan Province (Brain Hospital of Hunan Province), Changsha, China; ^3^Department of Integrated Traditional Chinese & Western Medicine, The Second Xiangya Hospital, Central South University, Changsha, China

**Keywords:** alcohol use disorder, gut microbiota, bibliometry, visualization analysis, research hotspot

## Abstract

**Background:**

Alcohol use disorder (AUD) is a psychiatric disorder that is widespread worldwide. Alcohol use is a significant contributor to the global burden of death, disability and disease. Modulation of the gut microbiota is a promising approach to improve the efficacy and minimize the adverse effects of colorectal cancer treatment. The relationship between the presence of microbes and AUD has been widely validated. However, few studies have examined this relationship using bibliometric methods. Therefore, this study analyzes the research hotspots and trends in human gut microbiology and AUD over the last two decades from a bibliometric perspective. This study aims at provide new directions for basic and clinical research in this field.

**Objective:**

A comprehensive discussion of the relationship between the current state of research and trends in AUD and intestinal flora.

**Methods:**

We collected publications from the Web of Science Core Collection database from 2003 to 2023 according to established inclusion criteria. We analyzed countries, institutions, authors, and research contributions using CiteSpace, VOSviewer, and Scimago Graphics to visualize research trends in the field.

**Results:**

A total of 2,102 publications were obtained, with a rapid increase in the number of publications since 2016. The United States and China are major contributors to the field and have established a network of partners in several countries. Five hundred ninety-five academic journals published articles on the topic. The author with the highest number of publications is Prof. Bernd Schnabl of the Department of Gastroenterology at the University of California, San Diego. In addition to “gut flora” and “AUD,” high frequency words in the keyword co-occurrence network analysis included alcoholic liver disease, tryptophan metabolism, enterohepatic axis, and fecal microbial transplantation.

**Conclusion:**

The results of this study provide a bibliometric analysis and visualization of key research areas in the gut microbiota and AUD over the past 20 years. The results suggest that the role of the gut microbiota in AUD and its potential mechanisms, especially therapeutic targets, should be closely monitored and could become a hot topic in the field.

## 1 Introduction

Alcohol use disorder (AUD) is a mental disorder that is widespread worldwide. Alcohol use is a significant contributor to the global burden of death, disability and disease. And with the 2019 Coronavirus Disease epidemic, the incidence of AUD is higher compared to past decades (Sarangi and Eskander, 2021). The global burden of disease research from 2020 according to due to alcohol, 3 million deaths worldwide are attributed to alcohol abuse each year, making up 5.3% of all fatalities (Shield et al., 2020). Alcohol use is predicted to increase globally, at least through 2030 (Manthey et al., 2019).

Drinking alcohol damages the intestinal barrier’s integrity, increases intestinal permeability, and modifies the intestinal flora’s composition. These modifications have a number of negative effects, including: central inflammation brought on by bile acid enterohepatic cycle impairment, intestinal bacterial dysbiosis, and alcohol-related mental illnesses (Wang et al., 2020). Probiotics have been demonstrated in numerous studies to increase beneficial bacteria in the gut microbiota, improving gut microbiota composition and improving both the physical and mental health of individuals with AUD (Wolstenholme, 2024). The human digestive tract’s lumen offers a physiologic home for a variety of bacteria, making the gut the body’s greatest reservoir of microbes (Meroni et al., 2019). The gut microbiota, primarily situated in the intestines, undergoes changes during growth influenced by physiological, pathological, dietary, and environmental factors (Qin et al., 2021). The makeup of the gut probiotic flora is closely related to human health. A dynamic equilibrium is perpetually maintained among various strains of gut microbiota, the host, and the surrounding environment, creating an ecosystem defined by interdependence and mutual constraints (Chang et al., 2023). The gut microbiota and its metabolites can regulate host behavior, metabolism, diet, and immune responses directly or indirectly through vagal and endocrine actions (Hillemacher et al., 2018; Dalile et al., 2019). Whereas chronic alcohol consumption leads to changes in the gut microbiome as well as changes in intestinal permeability, resulting in gut dysbiosis (Meroni et al., 2019; Litwinowicz and Gamian, 2023). Dysbiosis of gut microbiota can lead to intestinal inflammation, oxidative stress, and intestinal barrier damage (Hamer et al., 2007; Chen et al., 2015). Gut microbial metabolites, including lipopolysaccharide, peptidoglycan, lipoteichoic acid, and flagellin nucleic acids, can enter the bloodstream through the highly permeable intestinal barrier in AUD patients, causing systemic and neuroinflammation (Leclercq et al., 2012). Systemic inflammation and neuroinflammation are the main factors leading to the psychiatric symptoms of AUD (Qin et al., 2021). In addition, long-term alcohol consumption also alters the intestinal microbiome and leads to alcohol-related liver disease (ALD) by changing microbiota-derived metabolites, which is a hot topic for researchers, and researchers have focused on the intestinal microbiome for the treatment strategy and target of ALD (Mendes and Schnabl, 2020; Ranjbarian and Schnabl, 2023).

In the last decade, research has begun to focus on the link between the gastrointestinal microbiome and addiction. Chronic alcohol intake impairs intestinal barrier integrity and alters gut microbiota composition (Cryan et al., 2019; Meroni et al., 2019; Wang et al., 2020). Systemic inflammation and neuroinflammation due to alcohol-induced disruption of intestinal flora and the intestinal barrier are important factors in the development of AUD and psychiatric symptoms (Qin et al., 2021). When the gut microbiota of alcohol-dependent patients was transferred into rats, it was discovered that the gut microbiota caused changes in the behaviors linked to alcohol dependence in the rats, such as an increase in behaviors resembling anxiety and depression, a decrease in exploration and recognition memories, and an increase in alcohol preference (Wang et al., 2023). This bidirectional network facilitating information exchange between the brain and the gastrointestinal tract is referred to as the “brain-gut axis.” It plays a crucial role in neuronal development, brain function, cognitive regulation, and the aging process (Cryan et al., 2019). Prolonged alcohol intake causes microbial imbalance and intestinal permeability via the brain-gut axis pathway, which modifies the shape or function of specific brain regions and impacts memory (Li et al., 2022). In addition, alcohol can lead to mood recognition disorders and social cognitive dysfunction in adolescents (Carbia et al., 2020, 2023). In addition to the fact that alcohol can cause dysbiosis of the gut flora, which leads to cognitive impairment and mood disorders, researchers believe that dysbiosis of the gastrointestinal microbiome may cause the gut to transmit signals to the brain that promote addictive behaviors. The researchers concluded that individuals with pre-existing ecological disorders were more prone to addiction (Worth, 2024).

This research is the first bibliometric analysis to investigate the correlation between AUD and intestinal flora. It synthesizes two decades of literature on this topic, facilitating a comprehensive understanding of current advancements and future research trajectories in the interplay between gut microbiota and AUD. Bibliometrics utilizes mathematical and statistical techniques to analyze data from published sources (Chang et al., 2023). Such analyzes often encompass a substantial amount of objective data, enabling effective data visualization. This allows researchers to quickly grasp research trends within the field and to identify areas warranting further investigation (Doğan and Çelik, 2024). The aim of this study is to identify hotspots and trends related to gut flora and its influence on AUD over the past 20 years, thereby mapping the scientific landscape of this area, delineating its current status, and laying the groundwork for future research on the connections between gut microbiota and AUD.

## 2 Methods

### 2.1 Data source and search strategy

**Figure 1:**
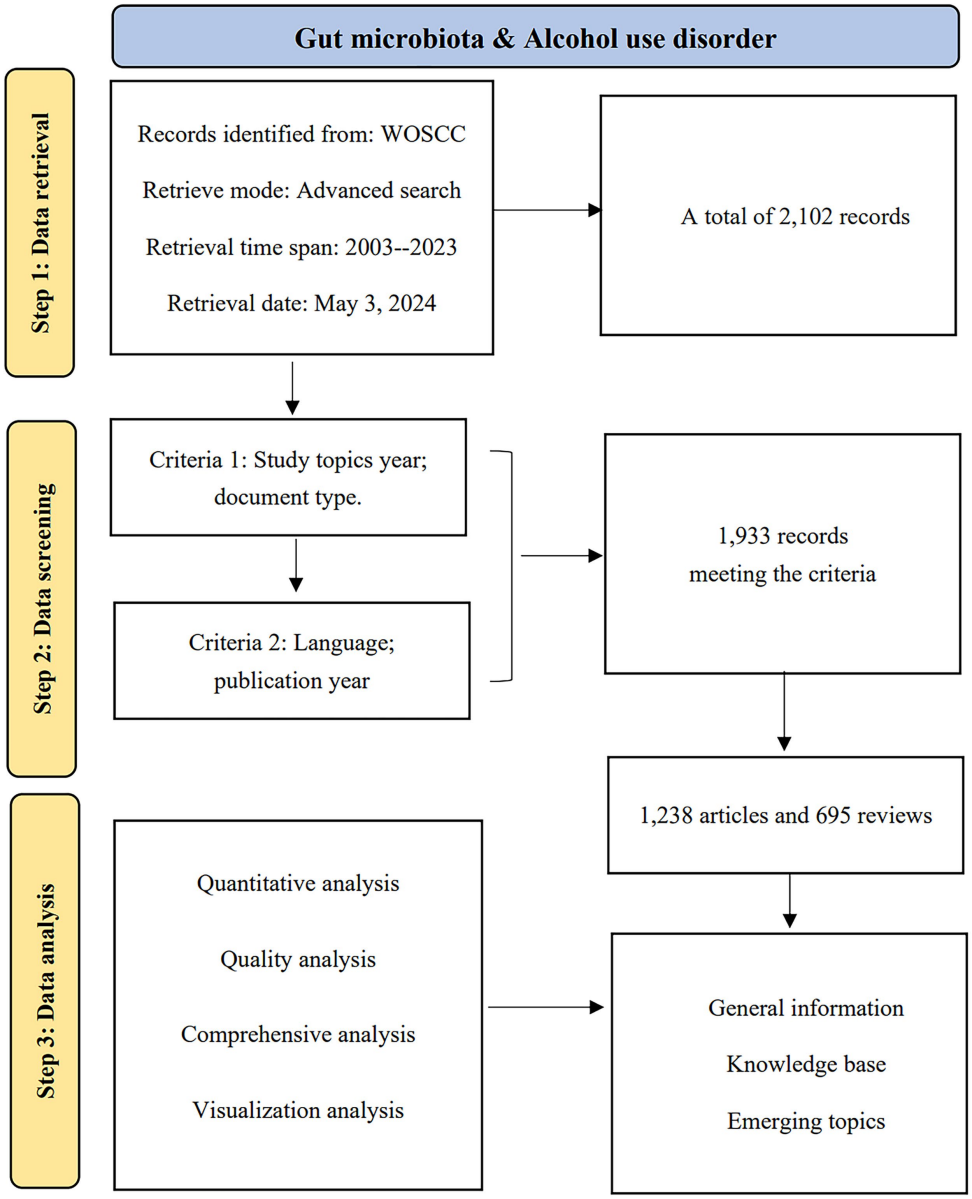
A flowchart of the publication screening process.

Data were sourced from the Web of Science (WoS) Science Citation Index (SCI) Extension database. To mitigate bias arising from daily updates to the database, all publications spanning from 2003 to 2023 were retrieved and downloaded on May 3, 2024, from the WoS Core Collection (WoSCC). The search strategy applied was Theme = (“Intestinal flora” OR “Gastrointestinal Microbiome” OR “Gastrointestinal Microbiomes” OR “Gut Microbiome” OR “Gut Microbiomes” OR “Gut Microflora” OR “Gut Microbiota” OR “Gut Microbiotas” OR “Gastrointestinal Flora” OR “Gut Flora” OR “Gastrointestinal Microbiota” OR “Gastrointestinal Microbiotas” OR “Gastrointestinal Microbial Community” OR “Gastrointestinal Microbial Communities” OR “Gastrointestinal Microflora” OR “Gastric Microbiome” OR “Gastric Microbiomes” OR “Intestinal Microbiome” OR “Intestinal Microbiomes” OR “Intestinal Microbiota” OR “Intestinal Microbiotas” OR “Intestinal Microflora” OR “Enteric Bacteria”) AND TS = (“alcohol use disorder” OR “alcohol abuse” OR “alcohol dependence” OR “alcoholi*” OR “alcohol addiction” OR “alcohol intoxication”). Figure 1 depicts the flowchart of the data screening process, which details the inclusion and exclusion criteria for the selected publications, as well as the analytical approach used.

### 2.2 Data analysis

CiteSpace (version 6.1), VOSviewer (version 1.6.18), Scimago Graphica (version 1.0.34), along with the open-source packages Bibliometrix and Biblioshiny, which operate within the R programming environment, were employed for both quantitative and qualitative analyzes, synthesis, and visualization of data. CiteSpace facilitated the examination of the most frequently cited references and keywords, generated timeline graphs, surveyed the current state of research, identified hotspots and emerging trends, and elucidated the dynamics within the field. Meanwhile, VOSviewer was utilized to visualize co-authorship relationships and the co-citation patterns of journals among authors. Scimago Graphica is used to visualize trends in collaboration between countries, as well as the extent and trends of collaboration between authors and their teams. R (version 4.1) is also used to visualize data analysis, including the top 10 cited authors, authors and journals with high *H*-indexes, and the distribution of topics and trends over the last 20 years. By examining the frequency of keywords and the trend of themes, we can get a full knowledge of the present research state, hotspots, and trends in the field.

## 3 Results

### 3.1 Annual trends in publications

**Figure 2:**
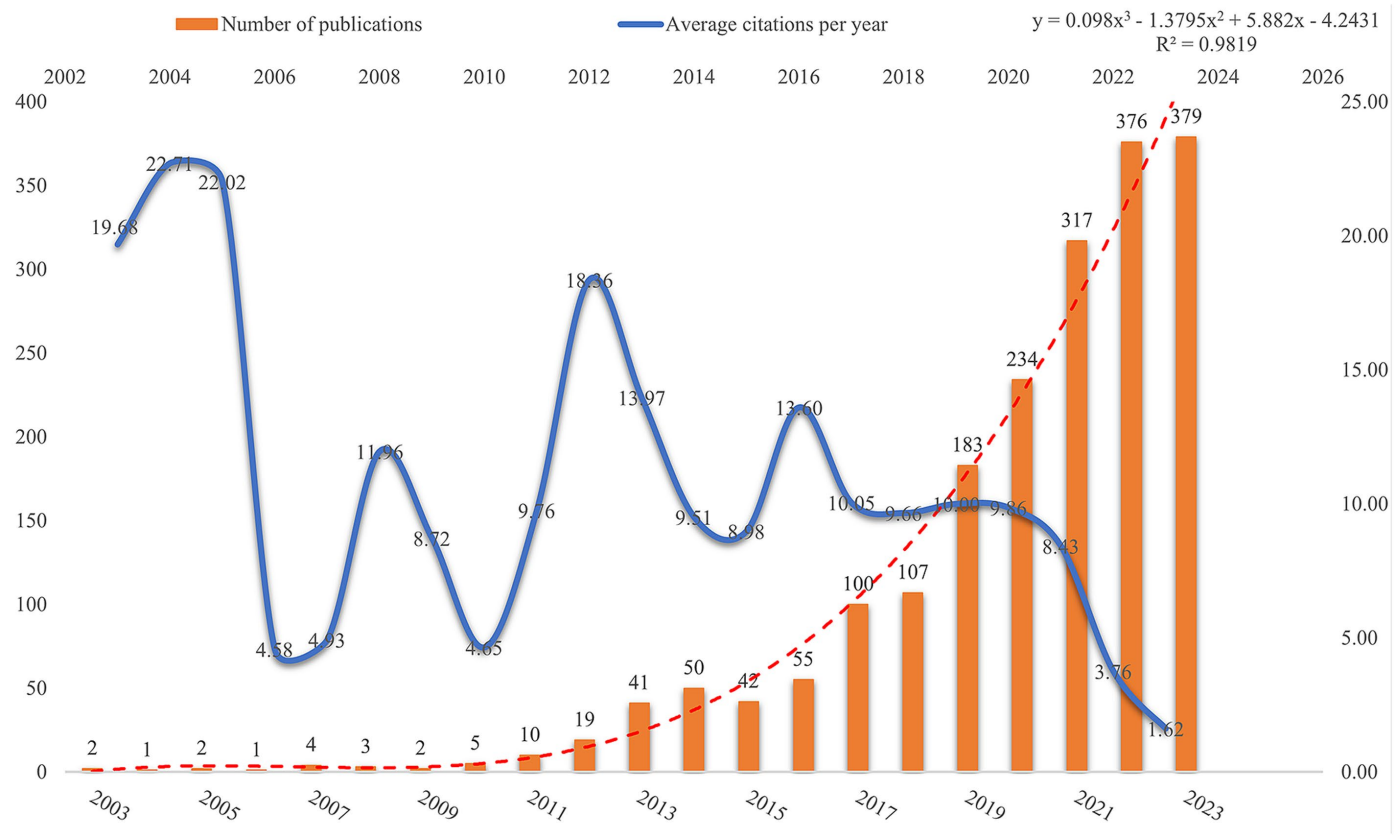
Trend distribution of articles on gut flora in the AUD field.

Publications in a field and trends in them can reflect its development stage and predict its growth. Based on the search criteria and time frame, WoSCC retrieved 1,933 publications related to AUD and intestinal flora, of which 1,238 (64.05%) were articles and 695 (35.95%) were reviews. Figure 2 illustrates the trend in annual publications concerning the gut flora associated with AUD from 2003 to 2023. This trajectory can be categorized into two distinct phases: a period characterized by gradual growth from 2003 to 2016, followed by an era of accelerated expansion from 2016 to 2023. Publications before 2016 grew relatively slowly, while publications after 2016 grew rapidly. Publications after 2016 are at least 100 per year, which shows an yearly growth trend compared to before 2016. AUD and gut flora publications reached 379 in 2023, which is the highest number in 20 years. Figure 2 presents a polynomial curve that depicts the annual increase in publications, demonstrating a strong correlation with the publication year (*R*^2^ = 0.9819). Collectively, these findings suggest a discernible trend of escalating research into the relationship between AUD and gut flora over the years. A growing number of researchers are actively exploring the role of gut flora in the context of AUD.

### 3.2 Analysis of countries and collaboration

**Figure 3:**
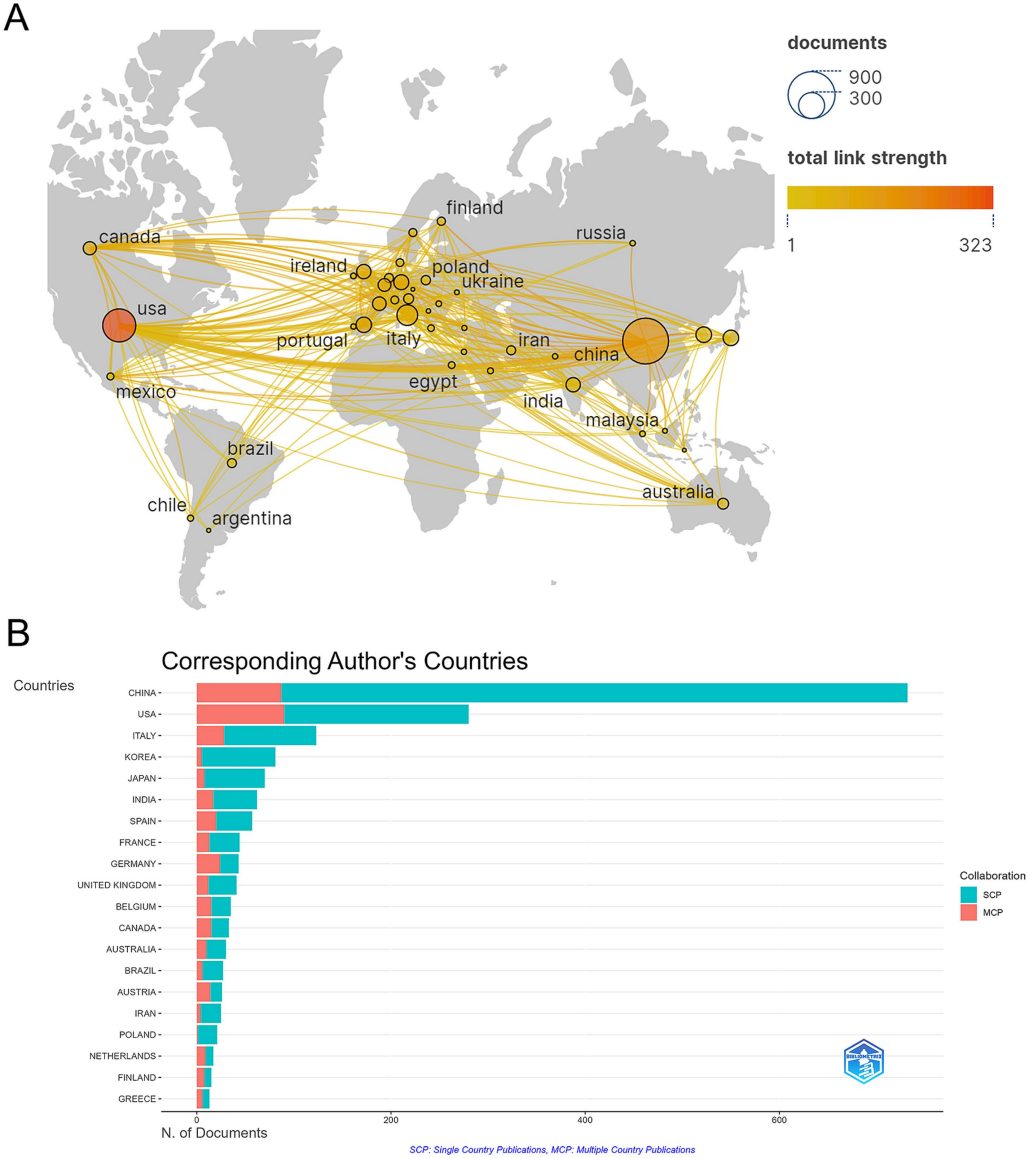
(A) Map of cooperation between states. (B) Visualization of the country of the corresponding author of a publication.

A total of 1,933 publications were released from 73 countries, indicating that research on gut flora and AUD has been conducted in several countries. China (732 papers), the United States of America (280 papers), Italy (123 papers), Korea (81 papers), and Japan (70 papers) are the top 5 countries in terms of publications (see Table 1). Among the 10 countries with the highest publication rates, Germany, Spain, and the United States exhibit notable levels of international collaboration, with 55.8, 50.7, and 50% of their contacts being international, respectively. This indicates a greater extent of global cooperation in their research efforts compared to China and Italy. To characterize cross-border cooperation, we imported the data into Scimago Graphics and Biblioshiny. The results are shown in Figures 3A,B. China has the most publications, but the United States has the most collaborations with other countries. Moreover, our analysis shows that AUD and gut flora cooperation between countries is close and frequent. Among the institutions, the University of California, San Diego emerged as the most prolific contributor, with a total of 69 publications. It was closely followed by Shanghai Jiao Tong University, which produced 42 papers, and Shanghai University of Traditional Chinese Medicine, with 41 papers. The VA San Diego Healthcare System and the Catholic University of Louvain also made significant contributions, with 38 and 37 publications, respectively, as illustrated in Table 2.

**Table 1:** Top 10 countries for publications.

Rank	Country	Articles	MCP	MCP_ratio
1	China	732	87	0.119
2	United States	280	90	0.321
3	Italy	123	28	0.228
4	Korea	81	5	0.062
5	Japan	70	8	0.114
6	India	62	17	0.274
7	Spain	57	20	0.351
8	France	44	13	0.295
9	Germany	43	24	0.558
10	United Kingdom	41	12	0.293

MCP, multinational publications; MCP_ratio, percentage of publications issued in international collaborations.

**Table 2:** Top 10 organizations with most publications.

Rank	Organization	Documents
1	University of California, San Diego	69
2	Shanghai Jiao Tong University	42
3	Shanghai University of Traditional Chinese Medicine	41
4	VA San Diego Healthcare System	38
5	Catholic University of Louvain	37
6	Zhejiang University	36
7	Jiangnan University	28
8	Hallym University	22
9	Huazhong University of Science and Technology	22
10	University of Naples Federico II	22

### 3.3 Authors and co-cited authors

**Figure 4:**
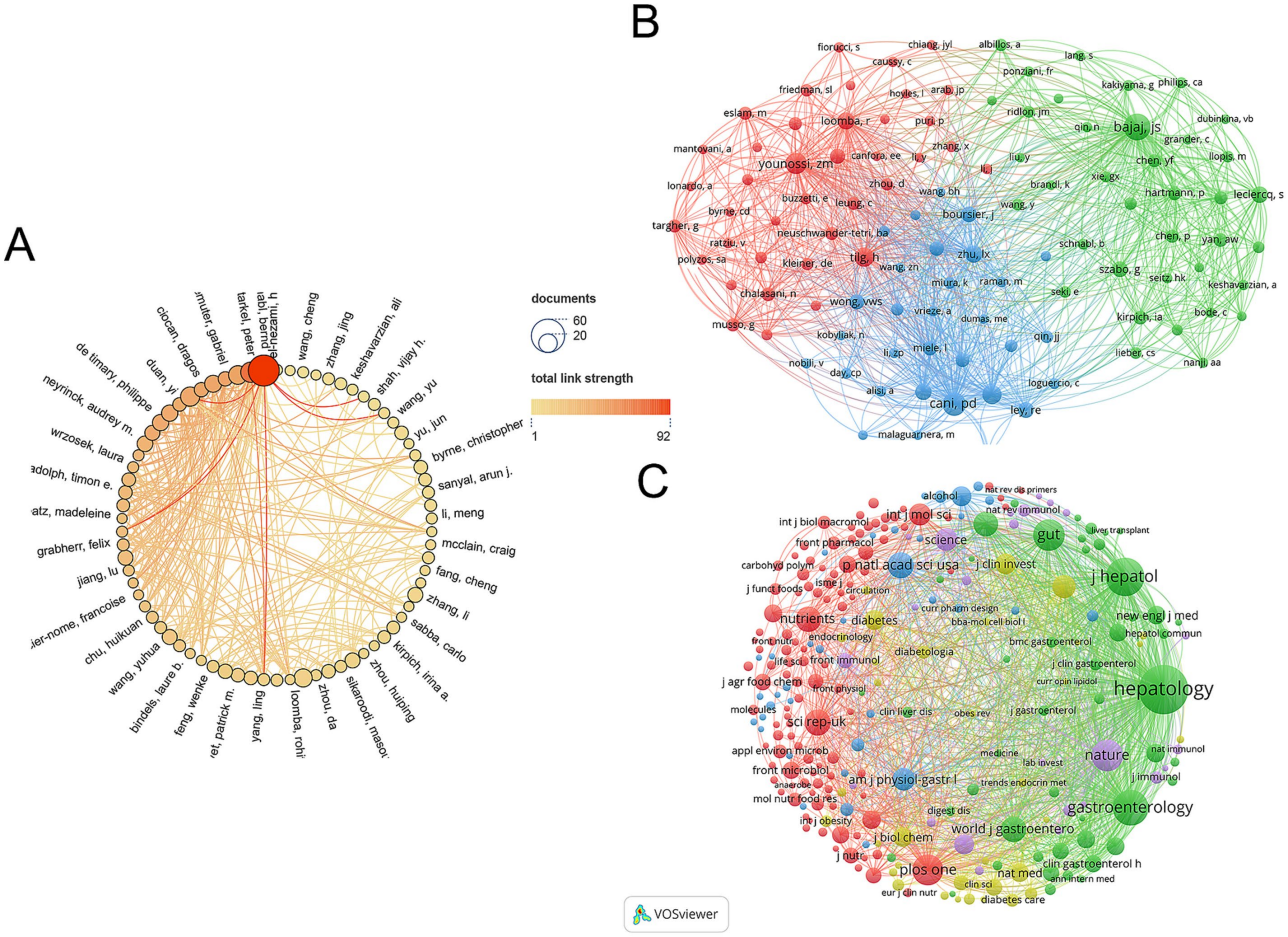
(A) Visual analytics for author collaboration. (B) Visual analysis of author co-citation. (C) Visualization of co-cited journals.

The gut microbiota of AUD was studied by 11,014 researchers according to an analysis of 1,933 publications. Figure 4A illustrates the Author Collaboration Network, where the size of each node corresponds to the number of publications, while the connecting lines signify the extent of collaboration. Larger nodes indicate a higher volume of publications, and thicker connecting lines reflect a greater degree of collaborative efforts. Bernd Schnabl, a professor of medicine in the Department of Gastroenterology at the University of California, San Diego, is the author of the most publications (47 papers) and has worked with many of the writers.

**Figure 5:**
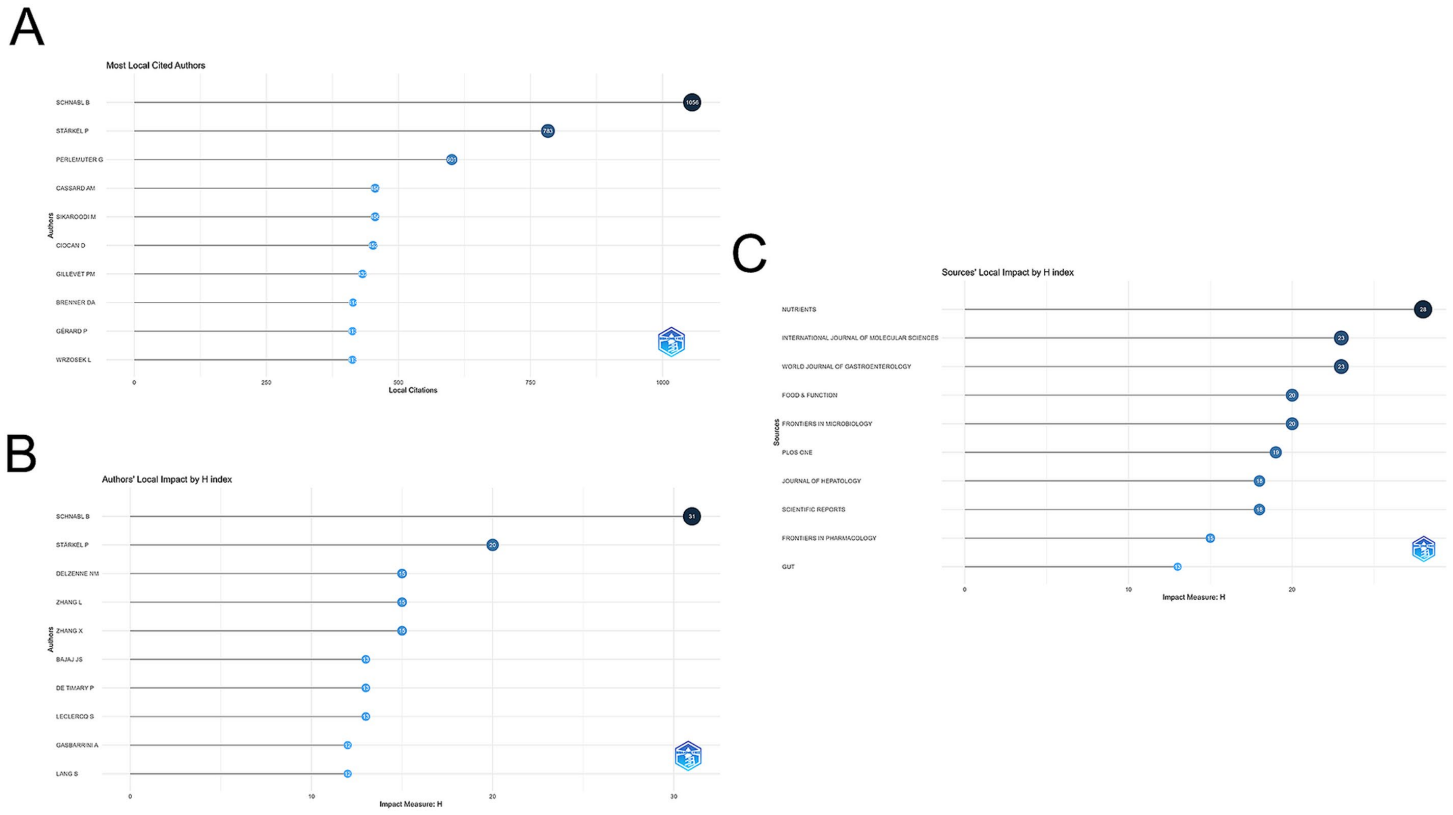
(A) Visualization of the 10 most cited authors using Biblioshiny. (B) Top 10 authors in the *H*-index. (C) Top 10 journals in the *H*-index.

VOSviewer is used to analyze and illustrate academic links among writers in a network. Co-citation refers to the phenomenon where two research articles are cited together in a third publication. As illustrated in Figure 4B, the size of each node corresponds to the number of citations attributed to the respective scholar. A greater number of connections between two nodes signifies a higher frequency of citations for both authors within the same work. Figure 5A lists the top 10 most co-cited authors. Schnabl’s B. publications have been cited 1,056 times, making him the most cited author.

### 3.4 Journals and co-cited academic journals

A total of 595 journals published AUD related to gut microbes, with Nutrients (97 papers, IF = 5.9) publishing the most, followed by International Journal of Molecular Sciences (88 papers, IF = 5.6) and Frontiers in Microbiology (54 papers, IF = 5.2). The Journal of Hepatology, while not the most published journal, has the greatest impact factor (IF = 25.7) and average amount of citations per publication (average citation/publication = 134.32) (see Table 3). As illustrated in Figure 4C, an analysis of co-cited journals was conducted to create a visual network that highlights the journals significantly influencing the advancement of this field. The size of each node correlates to the number of citations received, indicating that multiple journals have a positive citation connection with one another.

**Table 3:** Top 10 journals with most publications.

Rank	Source	Documents	Average citation/publication	IF
1	Nutrients	97	30.32	5.9
2	International Journal of Molecular Sciences	88	28.27	5.6
3	Frontiers in Microbiology	54	23.24	5.2
4	Food & Function	42	26.83	6.1
5	Frontiers in Nutrition	39	9.56	5.0
6	Frontiers in Pharmacology	39	33.46	5.6
7	World Journal of Gastroenterology	34	69.47	4.3
8	Scientific Reports	28	63.11	4.6
9	Frontiers in Endocrinology	23	22.39	5.2
10	Journal of Hepatology	22	134.32	25.7
10	PLoS One	22	71.95	3.7

### 3.5 Most cited publications and co-cited references

**Figure 6:**
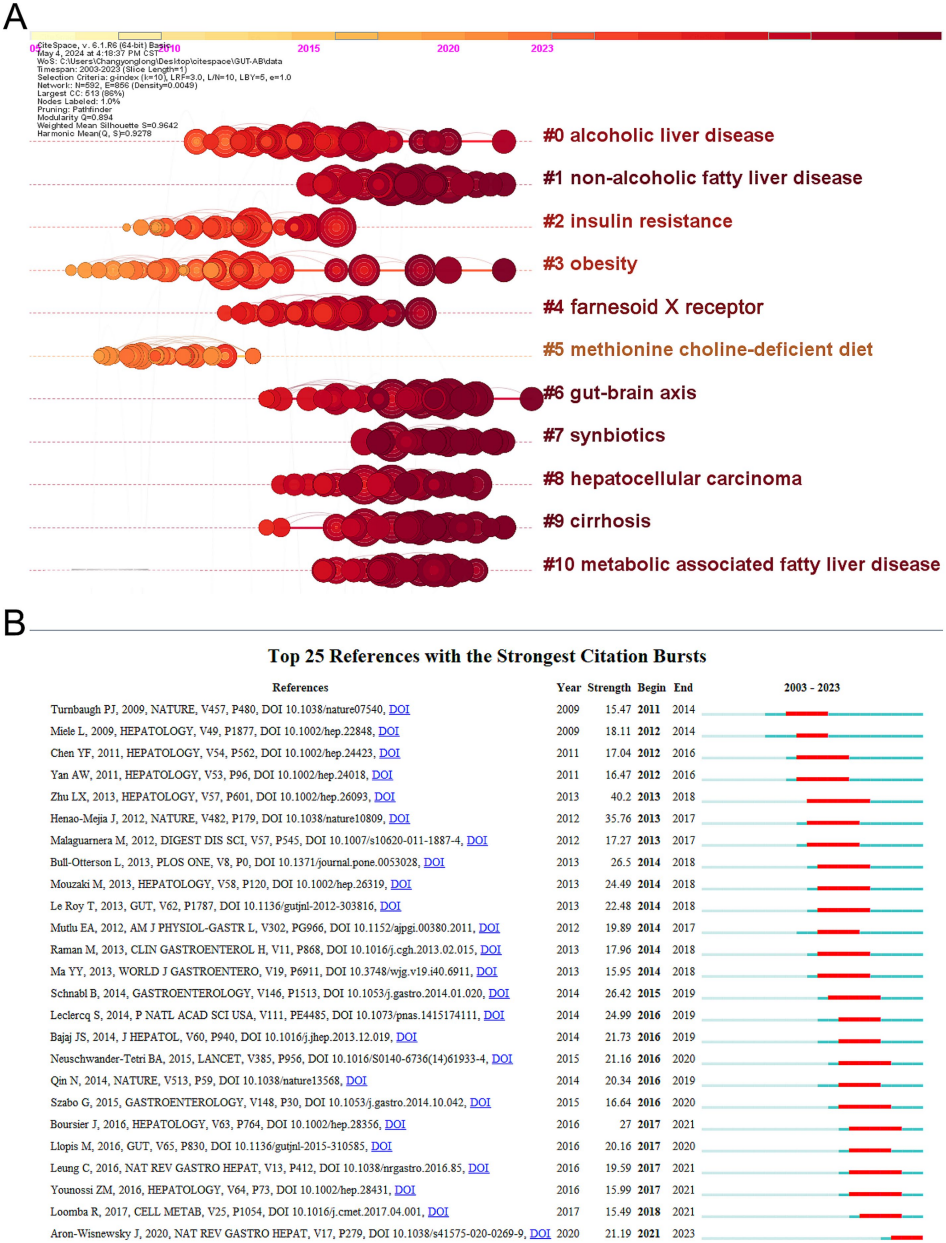
(A) Timeline visualization of co-cited publications. (B) Top 25 most cited references.

The application of CiteSpace to visualize timeline graphs of co-cited literature reveals trends and connections over time across different topics and groups in the field. As in Figure 6A, different colored nodes in the same row represent different years, different horizontal lines indicate different sets of cluster references, and cluster labels are located at the far right of the line. In the timeline graph, the closer the nodes are to the right represents more recent references. The closest cluster on the timeline is “#6 gut-brain axis.” In addition to this, researchers investigating alcohol and gut flora have also focused on “#0 alcoholic liver disease.” “#1 Non-alcoholic fatty liver disease,” also known as metabolic dysfunction related fatty liver disease, is a common condition marked by the buildup of fat in the liver in people who drink little or no alcohol (Hagström et al., 2024). The complicated connection between non-alcoholic fatty liver disease and gut microbiota has emerged as a major element in disease development and progression, and researchers are now focusing on it (Pi et al., 2024). The top 25 most cited references are depicted in Figure 6B, and it is evident that the initial citation surge occurred in 2011. A citation burst shows that the reference has been widely cited, and its conclusions are generally known and recognized in the field.

### 3.6 Analysis of co-cited keywords

**Figure 7:**
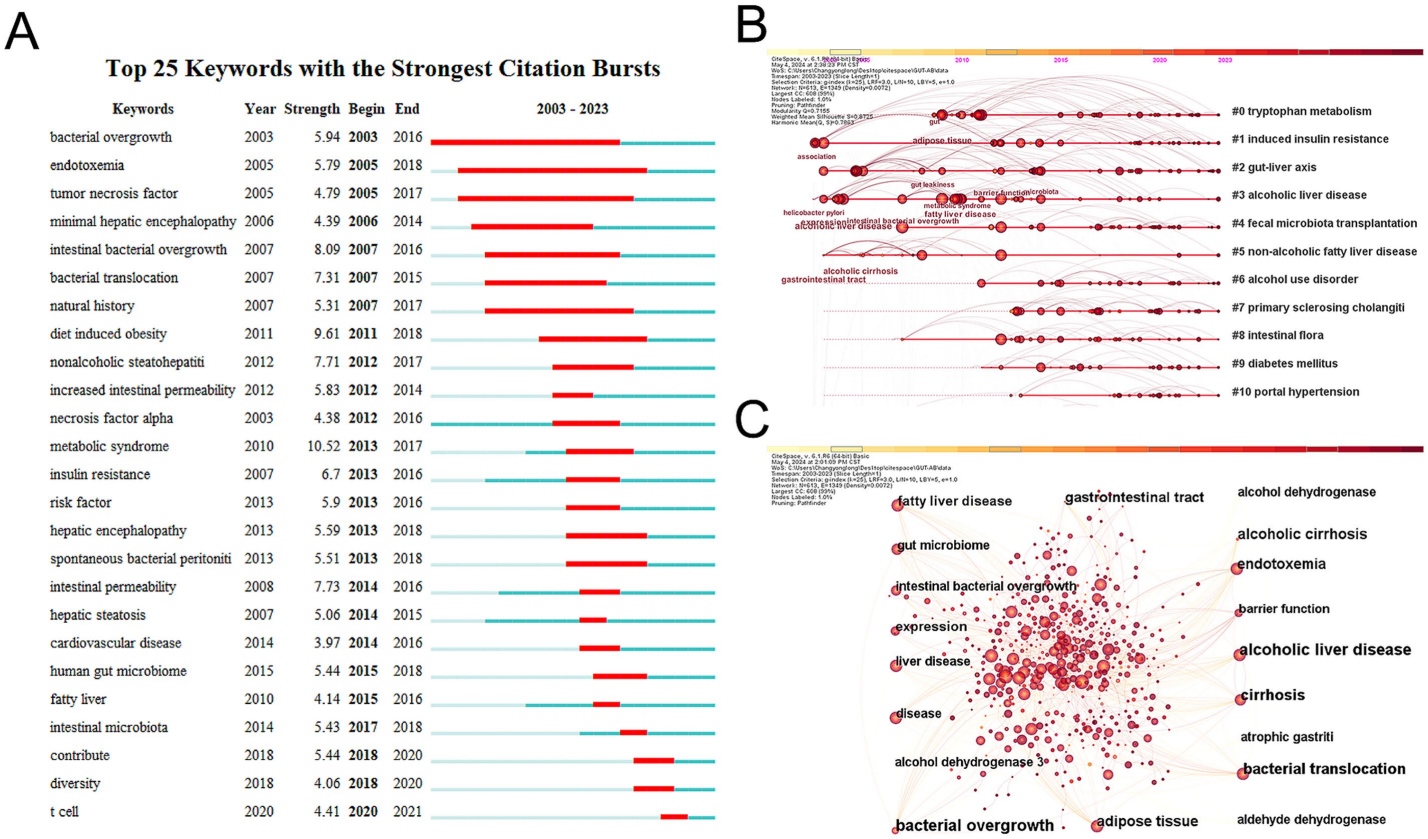
(A) Top 25 keywords with the strongest citation bursts in this field over the past 20 years. (B) Ten keywords timeline map. (C) Ten most frequent keywords for visualization.

Keywords describe the primary themes of the paper, and high-frequency keywords frequently reflect the hot study subjects in the area (Song et al., 2022). To construct a keyword co-occurrence network graph, a timeline of keywords, and a list of the top 25 terms exhibiting the highest citation intensity, we employed CiteSpace to extract and analyze keywords from a total of 1,933 articles. As shown in Figure 7C, we visualized these keywords. The top five clusters are #0 tryptophan metabolism, #1 induced insulin resistance, #2 gut-liver axis, #3 alcoholic liver disease, and #4 fecal microbiota transplantation, according to the keyword timeline plot in Figure 7B. Bacterial overgrowth, endotoxemia, and tumor necrosis factor have the greatest intensity values in the burst detection analysis in Figure 7A, suggesting that they were frequently cited within a certain time frame. These results show the broad interest in these terms and their correlation with field research hotspots.

### 3.7 Top 10 journals with the highest *H*-index scores

The *H*-index, proposed by Hirsch (2005), is a commonly used quantitative indicator that measures the productivity and effect of academic activity. The number of articles authored by a researcher with h or more citations and the remainder papers with h or fewer citations are indicated by the *H*-index. In this analysis, the leading journals that published research on gut flora in the AUD study were determined by using the *H*-index. As indicated in Figure 5C, Nutrients had the highest *H*-index, followed by the International Journal of Molecular Sciences and the World Journal of Gastroenterology. It’s interesting to note that, out of the top 10 publications, the World Journal of Gastroenterology ranked seventh for the quantity of articles published. However, it gained an impressive second place in terms of the *H*-index. This suggests that, while it may not have the largest publication volume, the works it has published are highly cited and significant in the field.

### 3.8 Top 10 authors with the highest *H*-index score

An extensively used quantitative metric for assessing the intellectual output of scholars is the *H*-index. Figure 5B depicts the top 10 authors based on *H*-index and overall citation frequency, with Schnabl B. having the highest *H*-index, followed by Stärkel P., and Delzenne N. M. and Zhang, L. tied for third place.

### 3.9 Thematic distributions and trends

**Figure 8:**
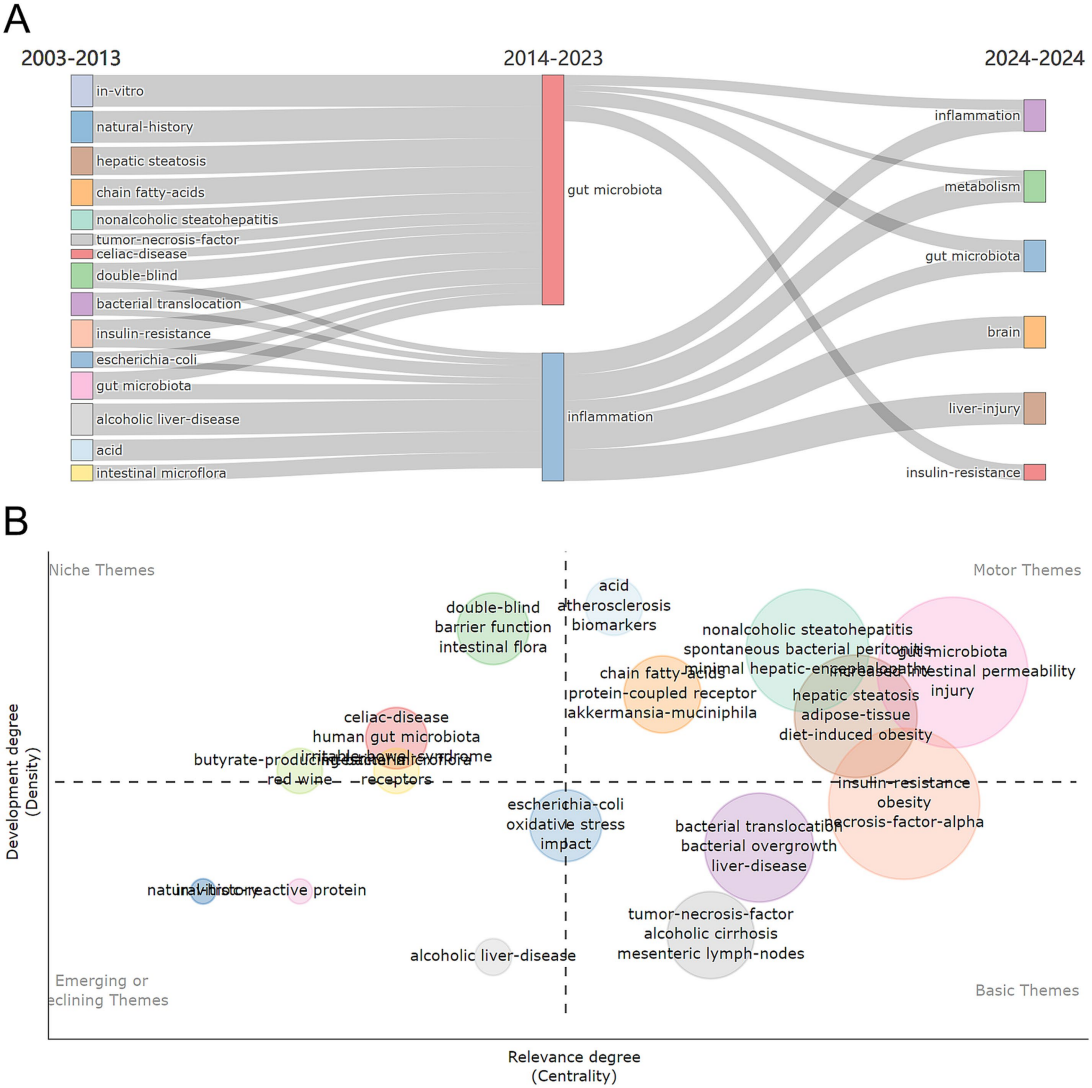
(A) Thematic evolution and map of research themes from 2003 to 2024. (B) Thematic map 2003–2024.

The density and centrality of individual research topics in AUD’s gut flora from 2003 to 2024 were visualized through thematic maps. The period is divided into two equal time slices, and it depicts the research landscape, providing a picture of current changes and emerging developments in the subject. Figure 8A presents the shift in research themes from 2003–2014 to 2014–2024: this flowchart illustrates how research themes have evolved over time. The left side covers 2003–2014, while the right side represents 2014–2024, demonstrating how particular subjects have evolved or become the focus of study.

Figure 8B depicts the thematic map, which divides the study themes into four sections depending on their developmental density and centrality. On the left side of the image, niche themes such as “barrier function,” “intestinal flora,” “double-blind” and “human gut microbiota” are niche themes that, while currently showing low centrality, their high development density suggests a high likelihood of evolving into movements or basic themes. They possess the potential to profoundly shape future research directions. The fundamental themes include “tumor-necrosis-factor,” “alcoholic cirrhosis mesenteric,” “lymphoma-nodes,” “bacterial overgrowth” and “bacterial translocation,” “bacterial overgrowth” and “bacterial translocation.” The fundamental themes represent ongoing research interests. These themes form the basis of the research field and have a broad and sustained scientific impact, supported by a large number of studies and citations. The themes “biomarkers,” “protein-coupled receptor,” “*Akkermansia muciniphila*” and “gut microbiota” constitute the motor theme. The motor theme represents current and popular research directions in this field of study. Emerging or declining concepts highlight emerging or declining concepts and their possible future relevance. “Alcoholic liver-disease” was considered as a theme for the emerging or declining category.

## 4 Discussion

In this study, we conducted a thorough analysis of publications on AUD and gut flora from 2003 to 2023 using bibliometric approaches and information visualization. Our study offers fresh perspectives in this field by conducting a thorough analysis of the existing literature. Our research indicates that the field of AUD and gut flora has been more important over the past 20 years, as evidenced by the rise in scientific papers in this area. Our study highlights the need for more research and advances our understanding of the subject.

Our study analyzes authorship, affiliation, country/region of origin, journals, keyword hotspots, co-citations, collaborations, and themes at three levels of quantitative, qualitative, and integrative analysis. We have also talked about how research themes in the discipline have evolved throughout time. Consequently, a thorough discussion of a number of topics is required in order to present our findings in their entirety.

### 4.1 General information

First, the total amount of publications each year from 2001 to 2016 remained relatively steady with little variation throughout. But after 2016, there was a sharp rise in the number of publications annually, which could be linked to the field’s advancement in scientific and technology research producing significant research findings. For example, with the increasing popularity of multi-omics integration technology, researchers have begun to combine metagenomics, transcriptomics, and metabolomics to comprehensively analyze the function of gut microbiota (Buffington et al., 2016; Perry et al., 2016; Schirmer et al., 2016). China has the most articles on AUD and intestinal flora over the previous 20 years, followed by the US and Italy, according to our review of those publications. University of California, San Diego was an institution with the most publications overall. The author with the most publications and the highest *H*-index is Bernd Schnabl, a professor of medicine in the Department of Gastroenterology at the University of California, San Diego. Among the journals, the Journal of Hepatology has the highest impact factor in the field, whereas Nutrients excels in publication volume and also possesses the highest *H*-index. Our data also highlights a discrepancy between impact and publication output; some journals with significant impact publish fewer articles, and conversely, some highly productive journals have lower impact factors. For example, although the Journal of Hepatology has a substantial impact, it publishes relatively few papers, suggesting that it prioritizes stringent quality control over its output.

### 4.2 Knowledge base

Eugene Garfield, an American intelligence scientist, first brought the notion of co-citation into the literature in 1973. Co-citation refers to the phenomenon where two or more publications are cited together in one or more later articles, signifying a strong relationship between those works. This link can highlight key research themes and the interconnectedness of scholarly contributions in a certain field (Shen et al., 2022). It is worth noting that although the primary focus of our study is the relationship between AUD and the gut microbiome, the bibliometric analysis also revealed literature related to NAFLD. This may be due to the overlapping mechanisms between NAFLD and AUD in gut microbiome research, particularly the imbalance of the gut-liver axis and abnormalities in fat metabolism. Therefore, the presence of NAFLD is not a deviation from the theme of our study, but rather is associated with the microbiome mechanisms discussed in the research. The temporal descriptions of co-cited papers indicated that the primary concern of researchers in AUD-related disorders was ALD. ALD encompasses a spectrum of hepatic conditions, including alcohol-related steatohepatitis, cirrhosis, and hepatocellular carcinoma. It is one of the most frequent chronic liver illnesses globally, accounting for almost half of all cases of cirrhosis (Teschke, 2019; Maccioni et al., 2021). Most alcohol is metabolized by the liver and other organs, disrupting the liver’s metabolic homeostasis, and causing alcoholic fatty liver disease in more than 90% of alcoholics (Guillot et al., 2019; Mackowiak et al., 2024). The pathogenesis of ALD includes hepatocyte death and regeneration, inflammation, intestinal dysfunction and ecological dysregulation (dysbiosis), ductular reaction and hepatic mitochondrial dysfunction. Gut dysfunction and dysbiosis are currently the ones being focused on (Mackowiak et al., 2024). Chronic alcohol consumption leads to gut dysfunction characterized by nutrient malabsorption, heightened intestinal permeability, diminished production of antimicrobial molecules, increased mucus thickness, a marked reduction in mucosal immune cells, and alterations in the gut microbiome (Maccioni et al., 2023). In preclinical models, the consumption of alcohol has been linked to gut dysbiosis and an overgrowth of intestinal bacteria (Hartmann et al., 2019; Mendes and Schnabl, 2020). In mice, chronic ethanol ingestion increases the numbers of anaerobic *Mycobacterium* and *Mycobacterium avium* but decreases the number of lower thick-walled bacterial phyla, which is associated with down-regulation of the expression of the genes for the antimicrobial drugs Reg3g and Reg3b (Yan et al., 2011). Prolonged alcohol use decreases the generation of antimicrobial peptides such as mucus and α-defensins, weakening the intestinal barrier and allowing endotoxins to reach the liver through the portal vein. This is a significant element in the etiology of ALD (Chen et al., 2015; Lee et al., 2021). Clinical research revealed both quantitative and qualitative changes in the fecal microbiota of people with alcohol use disorders and alcohol-associated liver illness, including an increase in *Mycobacterium avium* and a decrease in *Ackermannia* (Mendes and Schnabl, 2020). Significantly, in chronic alcohol-fed mice, reduction of the gut bacterial load decreased ethanol-induced steatohepatitis, maintained the intestinal barrier, and controlled subclinical intestinal inflammation (Hendrikx et al., 2018). Dysbiosis of the intestinal flora was associated with alcohol dependence and severity of cirrhosis, and worsening of dysbiosis was associated with progression of cirrhosis (Llopis et al., 2015). Liver damage and immunological activation are caused by gut dysbiosis and bacterial translocation. However, the development of multi-drug resistant bacteria is the main disadvantage of traditional antibiotic prophylaxis (Yan and Garcia-Tsao, 2016). As a result, reestablishing the gut’s ecological balance has both therapeutic and preventive applications. The gut microbiota can be altered to do this by phage therapy, symbiotic therapies, probiotics, prebiotics, fecal microbial transplantation, and dietary modifications (Duan et al., 2019; Litwinowicz et al., 2019; Shasthry, 2020). There is not enough proof to support their usage in clinical practice, and they all need more research.

The gut microbiota’s capacity to connect with the brain and so influence behavior has become an attractive notion in the realm of health and illness (Cryan et al., 2019). The gut microbiota interacts with the host, forming a crucial interaction that helps the body maintain homeostasis. Although each individual has distinct gut flora, there appears to be a certain balance that might bring health advantages (Rhee et al., 2009; De Palma et al., 2014). Therefore, if the needed gastrointestinal bacteria are reduced, the gastrointestinal, neuroendocrine, or immunological interaction will deteriorate, eventually leading to sickness as well as emotional and behavioral issues (Cryan and O’Mahony, 2011). Worth (2024) at Nature suggests that the gut microbiota may cause the gut to send signals to the brain that promote alcohol-addictive behavior. Numerous studies indicate that microorganisms can modulate the gut-brain axis, influencing various conditions and behaviors, including autism spectrum disorders, social interactions, anxiety, depression, dietary preferences, and food intake (Ntranos and Casaccia, 2017; Jang et al., 2018a, 2018b; Shan et al., 2020; Hayer et al., 2024; Joo et al., 2024). Leclercq et al. (2014) investigated the function of the microbiome in the development of alcoholism. Compared to alcohol-dependent patients with lower gut permeability, those with higher gut permeability experienced significantly more withdrawal-related anxiety and despondency in this study. Additionally, the intensity of despondency, anxiety, and cravings during withdrawal was significantly correlated with gut permeability levels. Recent research has discovered that transplanting the microbiota of human AUD patients into mice may reproduce some of the behavioral changes associated with alcohol dependence, such as decreased sociability, increased depression-like behavior, and increased stress levels (Zhao et al., 2019). It is possible that reduced ethanol production and lipolysis by specific bacterial genera is associated with reduced hepatic synthesis of β-hydroxybutyrate, thus preventing the neuroprotective effects of β-hydroxybutyrate (Leclercq et al., 2020). Decreases in protective species may negatively impact alcoholism by increasing intestinal permeability and psychiatric disorders (Day and Kumamoto, 2022).

### 4.3 Emerging topics

Keywords serve as vital indicators of research themes and core content. Analyzing co-occurrences of these keywords can illuminate the emergence and evolution of specific research themes. In this study, we employ CiteSpace to create a keyword clustering timeline graph, enabling us to identify the top 10 most cited keywords. Additionally, we will delineate the most frequently cited keywords to uncover the top 25 research outbreaks, thereby facilitating an exploration of the prevailing hotspots and trends at the intersection of AUD and intestinal flora.

High-frequency terms identified in the keyword co-occurrence network and the timeline view of keyword clustering (Figures 7A,B) include tryptophan metabolism (Leclercq et al., 2021) induced insulin resistance (Wickramasinghe et al., 2023) gut-liver axis (Mackowiak et al., 2024; Raya Tonetti et al., 2024) alcoholic liver disease (Dubinkina et al., 2017; Dukić et al., 2023; Litwinowicz and Gamian, 2023; Li et al., 2024) fecal microbiota transplantation (Day and Kumamoto, 2022; Lamas-Paz et al., 2024) and intestinal flora (Litwinowicz and Gamian, 2023; Worth, 2024). This indicates that gut flora and digestive-related diseases are prominent topics within the realm of AUD gut flora research (Mackowiak et al., 2024). Moreover gut flora significantly influences the onset progression and treatment of AUD (Day and Kumamoto, 2022; Litwinowicz and Gamian, 2023; Wang et al., 2023; Worth, 2024). The study of AUD intestinal flora has shifted from phenomenological to mechanistic studies to explore how immune metabolic and neural pathways of gut-brain communication impact AUD patients to discover potential therapeutic targets. In recent years the treatment of ALD and AUD-associated cognitive and emotional dysfunction with intestinal flora has been extensively studied. In a groundbreaking preclinical study, Ferrere et al. (2017) found that modulating the gut microbiome prevented the development of ALD. In this study mice raised and fed the same diet in two different institutions and the pathogenesis of alcohol-induced early ALD were compared. It was found that in ALD-sensitive mice the alcohol diet resulted in a decrease in the phylum Anaplasma ceci and Aspergillus and an increase in the phylum Actinobacteria and thick-walled bacilli. As a result there were 50% more *Mycobacterium avium* in ALD-resistant mice than in ALD-sensitive mice. For further validation feces from ALD-resistant mice were transferred to ALD-sensitive mice for fecal microbial transplantation and it was found that both pectin and fecal microbial transplantation protected the mice against alcohol-induced liver lesions and maintained intestinal homeostasis. Subsequent investigations into the potential of probiotics or dietary supplements that alter microbiota to mitigate ALD symptoms were conducted after this study and the results generally showed that probiotic therapy improves liver protection (Tian et al., 2020; Gupta et al., 2021; Wolstenholme, 2024). Furthermore *Bifidobacterium longum* NCC3001 suppressed reactions to unpleasant emotional stimuli in many brain regions and decreased patients’ depression ratings on the Anxiety and Depression Scale by more than 60% (Pinto-Sanchez et al., 2017). *Lactobacillus plantarum* DR7 reduces anxiety symptoms and enhances cognitive and memory impairment. *Lactobacillus plantarum* 299V improves cognition by affecting the kynurenine pathway (Chong et al., 2019). It is therefore concluded that gut flora is a treatment for the potential of AUD. Consequently further investigation into the efficacy of probiotics prebiotics and fecal microbiota transplantation as potential treatments for this condition is warranted.

In conclusion, research over the past two decades on gut flora in AUD has identified five primary categories, as revealed by clustering analyzes of keyword co-occurrence networks and timeline graphs. These categories encompass: the roles and mechanisms of gut flora in ALD, the impacts on individuals with AUD, the influence of the brain-gut axis on AUD and its mechanisms of action, and, finally, the potential of gut flora as an innovative therapeutic strategy for both AUD and ALD (Li et al., 2022; Wolstenholme, 2024).

## 5 Limitations

One of the study’s weaknesses is that, while we focused on the association between AUD and gut microbiota, NAFLD was also included in the bibliometric analysis due to cross-over mechanisms. Future studies can separate the independent association between different types of liver illnesses and gut microbiota, as well as elucidate the specific mechanism. The study also shows some limitations inherent in bibliometric analysis. Although visual analytical tools offer a more sophisticated comprehension of the development of research goals and trends about the association between gut flora and AUD than conventional methods, the incorporated literature may not be exhaustive. Our analysis was based solely on the core dataset from the Web of Science database, which means that data from other significant search engines and journals not indexed in WoS-compliant sources were excluded. Additionally, focusing exclusively on English-language literature led to the exclusion of non-English articles that may hold valuable insights. In future research, we plan to address this limitation by manually integrating data from various databases or employing advanced data processing techniques when feasible. This approach aims to compile a more comprehensive dataset, ultimately strengthening our conclusions and providing valuable information and support to other researchers in the field.

## 6 Conclusion

In the field of AUD, gut flora presents significant therapeutic and research potential. We conducted a thorough econometric and statistical analysis of the intestinal flora research conducted in AUD over the past two decades in this study. We examined 2,102 publications from 2003 to 2023 using visualization tools such as CiteSpace, VOSviewer, and Scimago Graphica. Our findings indicate a significant increase in the volume of publications in leading international journals, with the United States and China emerging as the foremost contributors. Furthermore, researchers from various countries and institutions are actively collaborating in this area. Future research will likely emphasize the use of probiotics and the exploration of gut flora as therapeutic targets for AUD. Future research should concentrate on the integration of these discoveries into the clinic, thereby facilitating the transition from laboratory research to real-world patient care and offering patients a greater variety of and more effective treatment protocols.

## Data Availability

The raw data supporting the conclusions of this article will be made available by the authors, without undue reservation.
